# The effectiveness of neuromuscular electrical stimulation on pain, function, and quadriceps muscle strength in adults with patellofemoral pain: A systematic review and meta-analysis

**DOI:** 10.1186/s12891-025-09029-5

**Published:** 2025-08-09

**Authors:** Ahmed Ibrahim Abdelhamed, Hossam Mortada, Ahmed Hendawy, Amr Elfeky, Roshdy M. Kamel, Shorouk Elshennawy

**Affiliations:** 1 Faculty of Physical Therapy, Misr University for Science and Technology, 6th of October, Giza, Egypt; 2 Biomechanics Unit, Basic Sciences Department, Faculty of Physical Therapy, Galala University, Suez, Egypt; 3 Department of Physical Therapy for Neuromuscular Disorders and Surgeries, Faculty of Physical Therapy, Galala University, Suez, Egypt; 4 Faculty of Physical Therapy, Cairo University, Giza, Egypt; 5 Physical Therapy Department, Faculty of Allied Medical Sciences, Al- Ahliyya Amman University, Amman, Jordan; 6 Basic Sciences Department, Faculty of Physical Therapy, Benha University, Qalyubiyya, Egypt; 7 Pediatric Physical Therapy Department, Faculty of Physical Therapy, Cairo University, Giza, Egypt; 8 Pediatric Physical Therapy Department, Faculty of Physical Therapy, Misr University for Science and Technology, Giza, Egypt

**Keywords:** Patellofemoral pain, Neuromuscular electrical stimulation, Quadriceps muscle strength, Pain

## Abstract

**Background:**

Patellofemoral pain (PFP) is a chronic musculoskeletal disorder, with Neuromuscular electrical stimulation (NMES) often used as a complementary treatment option, however no definitive evidence supports its benefits. This systematic review aimed to evaluate the efficacy of adding NMES to exercise programs for treating PFP.

**Methods:**

PubMed, Cochrane CENTRAL, Web of Science, Scopus, PEDro, ClinicalTrails.gov and International Clinical Trials Registry Platform were searched up to July 2024 for randomized controlled trials (RCTs) investigating the effectiveness of NMES in people with PFP. Two researchers independently screened titles and abstracts; another pair filtered full-text articles. The studies had to report pain, functionality, and strength as outcome measures. The risk of bias was assessed using the revised Cochrane Collaboration tool (RoB 2.0). Meta-analysis was conducted using RevMan 5.4.1 software, and the quality of evidence was evaluated using the GRADE approach.

**Results:**

Eleven RCTs, involving 571 participants, were included. Most of the studies were of high risk of bias. NMES combined with exercise was favored in the long course for improving function (SMD: 3.74, 95% CI 1.35, 5.81; *P* = 0.0004) and quadriceps muscle strength (SMD: 0.53, 95% CI 0.18, 0.89; *P* = 0.003) compared to exercise; however, it did not differ from exercise in relieving pain and improving EMG activity level, and participation. The GRADE certainty rating of the results was rated as very low mostly due to inconsistency and high risk of bias.

**Conclusion:**

Very low certainty of evidence suggests that adding NMES to an exercise program may improve the knee joint function and quadriceps strength with treatment courses of six weeks or more in patients with PFP, but it is not superior to control for reliving pain and improving EMG activity, activity level, and participation. Larger, high-quality RCTs on the long-term are needed to reach an incisive conclusion.

***Registration:*** The protocol was registered at Open Science Framework Register under 10.17605/OSF.IO/Y2JUZ.

**Supplementary Information:**

The online version contains supplementary material available at 10.1186/s12891-025-09029-5.

## Introduction

Patellofemoral Pain (PFP) refers to anterior knee pain caused by pathological or biomechanical abnormalities. It can be exaggerated by functional activities, such as running, squatting, stair climbing, jumping, or prolonged sitting with a flexed knee [1]. It is reported to occur in 22.7% of the general population, and in 28.9% of adolescents, with a higher prevalence in athletes and military recruits [2].

It was theorized that patellar malalignment may be the main cause of anterior knee pain. Additionally, other causes, such as mechanical and structural changes in the patellofemoral joint, weakness of the knee extensor muscles i.e., quadriceps, poor muscle flexibility, and altered lower extremity kinematics have been linked to PFP [3]. In addition, activities with increased flexion angle, excessive adduction and/or internal rotation of the hip joint and excessive foot pronation are associated with higher stresses in the patellofemoral joint which may lead to the presence of patellofemoral pain [4–6].

Treating PFP may be quite challenging, and it may include one or several measures, such as conservative treatment, pharmacotherapy, and/or surgical treatment depending on the complexity of the case. Physiotherapy is considered the gold standard method in treating PFP. Many conservative methods or combinations of methods, such as closed kinetic chain exercises, open kinetic chain exercises, Kinesio taping, electrical muscle stimulation, patellar bracing, and/or foot orthotics have been proven to be significant results in treating patients with PFP [7]. Surgical treatment is considered as a last resort in PFP treatment; however, its efficacy is arguable as some authors inferred that it does not add any additional benefits [8].

Electrical stimulation is one of the adjunctive methods used to treat PFP. It can be used with other interventions, such as exercises or cryotherapy, or as a single treatment to promote muscle activation and improve muscle contraction and recruitment [3].

Neuromuscular electrical stimulation (NMES) is a type of electrical stimulation that is used to induce passive muscular contraction. It is widely used in clinical settings with a variety of neuromotor disorders as it is a passive, pain-free and non-invasive method of strengthening muscles [1]. NMES can directly depolarize motor axons, leading to involuntary contraction [9]. Russian and high voltage pulsed currents are popular neuromuscular electrical stimulation (NMES) methods for strengthening. These modalities are favored due to their high tolerance and minimal pain levels [10, 11].

As previously mentioned, muscle weakness is considered one of the major contributors to the development of PFP, therefore, several clinical trials have been investigating the effects of adding NMES to traditional physical therapy programs in the management of PFP [1, 3].

Despite the widespread use of NMES in clinical settings for neuromotor disorders, its role as an adjunct to traditional physical therapy for PFP remains uncertain due to conflicting findings in the literature. Some studies report that NMES, when combined with exercise, significantly improves pain, muscle strength, and knee function compared to exercise alone [1, 20]. For instance, trials have shown enhanced quadriceps strength and reduced pain scores with NMES, particularly in long-term treatment protocols [12]. Conversely, other studies have found no significant benefit of adding NMES, suggesting that traditional physical therapy alone may be sufficient for managing PFP symptoms [17, 18].

The inconsistent findings in the literature highlight a critical gap in understanding the clinical utility of NMES as an adjunctive therapy for PFP. Previous reviews have focused broadly on conservative treatments for PFP but have not specifically addressed the added value of NMES or systematically synthesized its effects across key outcomes [3]. This lack of clarity limits evidence-based recommendations for clinicians and underscores the need for a comprehensive evaluation of NMES’s effectiveness.

The objective of this systematic review and meta-analysis is to evaluate the effectiveness of adding neuromuscular electrical stimulation (NMES) to traditional physical therapy compared to traditional physical therapy alone in improving pain, muscle strength, and knee function in individuals with Patellofemoral Pain (PFP), thereby addressing the current gap in understanding its clinical significance.

## Methods

This review was registered in open science framework registries (OSF) under DOI 10.17605/OSF.IO/Y2JUZ on 21st August 2024. The authors adhered to the Preferred Reporting Items for Systematic Reviews and Meta-Analysis (PRISMA 2020) checklist [12].

### Search strategy

A Comprehensive systematic search was done by two researchers independently (AA, AE) in the following databases: PubMed, Scopus, Web of Science, PEDro and Cochrane Central. These databases were searched from their inception to July 2024. Manual search for studies was done to ensure search accuracy, detailed search strategy is available at appendix 1.

### Eligibility criteria

The prespecified Inclusion criteria for the reports included in this systematic review were as follows; randomized controlled trails investigating the efficacy of NMES added to traditional physical therapy programs such as exercise compared to physical therapy alone in patients with PFP on sensorimotor outcomes such as pain, activity, muscle strength or function.

Reports were excluded if they were non-peer reviewed articles or published only as conference abstracts or papers, in addition, reports written in any language other than English were not eligible for inclusion.

### Records screening

Title and abstract filtration were done independently by two researchers (AIA, HM) using Mendeley reference management software (Elsevier, Amsterdam, The Netherlands). Abstracts meeting inclusion criteria or those that required more information to determine their eligibility were retained for full-text review. Full Text filtration was done by two researchers (AE, AH) based on the pre-specified eligibility criteria.

### Risk of bias assessment

The eligible reports went through risk of bias assessment by two authors (HM, AIA) using the Cochrane risk of bias assessment tool (ROB 2.0) and the risk of bias assessment charts were created using the Risk of Bias visualization tool (ROBVIS) [13, 14], in addition, evidence emerging from outcomes that were included in the pooled statistical analysis were graded using the Grading of Recommendations Assessment, Development and Evaluation (GRADE) [15]. Conflicts between the two assessors were solved by a third assessor (SE).

### Data extraction

Population characteristics, Intervention parameters, outcome measures, and results were tabulated by an author (AE) and reviewed by another author (AH) to ensure reliability.

### Statistical analysis

We analyzed data from the included studies using Review Manager (RevMan– version 5.4.1, The Nordic Cochrane Center, The Cochrane Collaboration, Copenhagen, Denmark, 2021), and Microsoft Excel 2019 (Microsoft Corp., Redmond, WA, USA). A formal meta-analysis was conducted for all outcomes if the data were sufficient.

We expressed pooled effect measures as the mean difference (MD) with 95%CI. In cases where included studies used different scales of measurements, we used the standardized mean difference (SMD) instead. For studies reporting median and interquartile range (IQR), we used the median as an expression of the mean and we estimated the SD by dividing the IQR by 1.35 [16].

We explored and quantified between-study statistical heterogeneity using the I^2^ test. By default, we used the fixed effect model in all analyses. If heterogeneity was statistically significant (*p* < 0.05) or I^2^ was > 50%, we used the Der Simonian and Laird random-effects model instead [17]. We considered 2-sided statistical analysis testing, setting the α-error level at 0.05.

## Results

**Fig. 1:**
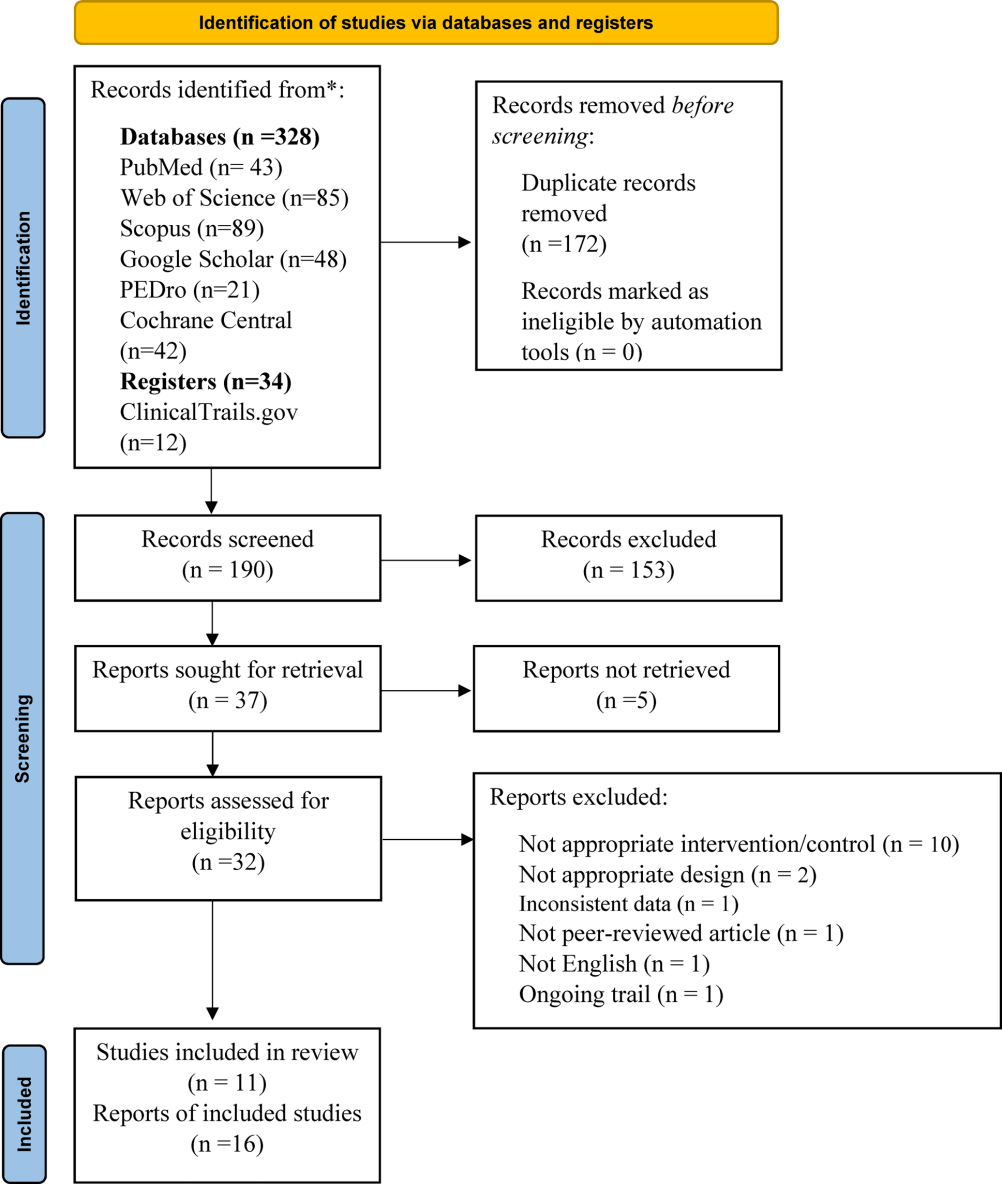
PRISMA flow diagram Source: Page et al., 2021 [12]

A systematic search yielded 362 records, which were screened after removing duplicates, resulting in 190 unique records. Thirty-two full-text articles were assessed for eligibility, and 11 studies [1, 18–27], represented by 16 reports [1, 18–32], met the inclusion criteria Figure 1 PRISMA flow diagram [12]. We excluded 14 studies with a total of 16 reports; the reasons for exclusion are detailed in Appendix (2).

All included studies were randomized controlled trials (RCTs) involving 571 participants diagnosed with PFP with an average age range of 22 to 46 years. Nine studies applied NMES with low-frequency [1, 18, 19, 21–23, 25–27] while two studies utilized medium-frequency NEMS [20, 24]. NEMS was applied in conjunction with exercise in all but one study, where it was combined with both exercise and patellar taping [25]. Electrode placement varied, targeting the quadriceps muscle [18, 19, 21, 25–27], quadriceps and gluteus medius muscles [20, 24], or the vastus medialis oblique (VMO) and gluteus maximus muscles [1].

Two studies employed Patterned Electrical Neuromuscular Stimulation with stimulation patterns targeting both agonist muscles (VMO and gluteus medius) and antagonist muscles (hamstrings and adductors) [22, 23]. The control groups in the included studies received either exercise alone [1, 18–21, 26, 27], exercise with sham NEMS [22–24], or exercise with patellar taping [25] Table 1.

The duration of NEMS application varied from 3 to 12 weeks, with frequencies ranging from 2 to 10 sessions per week, each lasting between 10 and 20 min. Follow-up periods ranged from 1 week to 1 year [20, 21, 23, 24, 26, 27].

Outcome measures across studies included pain, function, muscle strength and activity, kinematic analysis, activity and participation and quality of life. Whenever possible, data were pooled across two assessment time-points: short-term treatment courses i.e., less than 6 weeks and long-term treatment courses i.e., 6 weeks or more.

**Table 1:** Characteristics of the included studies

Study ID	Population		Intervention			Outcome Measure		Follow up	Drop out	Results
	N (S:C)	Age	S	C	Duration					
						Domain	Tool			
Nie et al., 2024	13:13	34.58	NMES for QC and GMax F: 20 Hz PD: 200 μs DC: 1:2 D: 20 min + exercises	Exercises program	Three sessions/week, six weeks	Pain	VAS10	NA	Two	There was a statistical significance in favor of SG regarding pain intensity and Knee function (P = 0.02 and 0.02) respectively Regarding muscle activity, VMO and VL activity showed no statistical difference between groups either in RMS or iEMG while VMO/VL ratio showed higher values in the SG in both the RMS and iEMG (P = 0.01 and 0.01)
						Function	KPS			
						Muscle Activity	EMG			
Talbot et al., 2020	30:34	26.7	NMES for QC F: 50 Hz PD: 300–400 μs DC (on/off): 5s/10s D: 20 min + Home exercises program	Home exercises program	Three sessions/week, nine weeks	Pain	VAS10	NA	NA	Statistically significant differences were found in favour of NMES group regarding knee flexion and extension strength while no difference were found regarding pain intensity relative to baseline scores over 9 weeks
						Muscle Strength	HHD			
Kumar et al., 2023	31:30	29.4	NMES on QC F: 20–50 Hz DC (on/off): 1:3 PD: 150–300 μs D: 20–30 min + exercises	QC strengthening exercises	Three sessions/week, 10 weeks	Pain	VAS10	NA	NA	Two-way ANOVA revealed statistical significance in favor of SG (P < 0.001, respectively) for KPS, but not VAS (P = 0.672)
						Function	KPS			
Melo et al., 2024	17:17	23.8	NMES on VMO and GMed F: 2500 Hz with 50 Hz burst PD: 200 μs DC (on/off): 1:1 D: 11 min	Strengthening exercises	Two sessions/week, eight weeks	Pain	PPS	Four months	Five	There is no statistically significant difference between groups in PPS, KPS, and EMG (P > 0.05)
						Function	KPS			
						Muscle Activity	EMG			
Celik et al., 2020	14:13	40.3	NMES on QC F: 50 Hz PD: 400 μs DC (on/off): 10:20 D: 20 min + exercises program	Exercises program	Three sessions/week, six weeks	Muscle Strength	Isokinetic dynamometer	Three, six and twelve weeks	Seven	There is no statistically significant difference between groups in KPS, Hamstring strength, and Quadriceps strength (P = 0.97, 0.29 and 0.37) respectively
						Function	KPS			
Glaviano et al., 2020	8:8	23.3	PENS F: 50 Hz PD: 200 ms stimulus train D: 15 min + exercise program	Sham PENS + exercise program	Three sessions/week, four weeks	Pain	VAS10	NA	Zero	The article did not report between group statistical comparison however, manual calculation reveals no statistical difference between groups regarding pain intensity (p = 0.9)
						Muscle Activity	EMG			
Glaviano et al., 2019	11:10	23.46	PENS F: 50 Hz PD: 200 ms stimulus train D: 15 min + exercise program	Sham PENS + exercise program	Three sessions/week, four weeks	Pain	VAS10	Six months and one year	one	No group main effect or group-by-time interactions were identified on the KPS, VAS or muscle strength
						Function	KPS			
						Muscle Strength	HHD			
Çankaya et al., 2024	16:18	36.1	NMES (Russian) on QC and GMed F: 2.5 kHz with 50 Hz burst frequency PD: 200 μs DC: 50% D: 10–15 min + 30–45 min Isokinetic training exercises	Isokinetic training exercises for 30–45 min	Five session/week, three weeks	Pain	PPSC	Three weeks	Three	No difference between treatment groups regarding all outcome measures post-intervention
						Function	KPS			
Kaya et al., 2013	15:15	43.9	HVPGS F: 60 Hz PD: 65–75 μs D: 20 min + patellar taping and exercises program	Patellar taping and exercises program	Five sessions/week, six weeks	Pain	VAS100	NA	Zero	statistical significance in favor of SG (P < 0.02) for VAS during step-up, but not VAS step-down or LEFS (P = 0.11, 0.79) respectively
						Function	LEFS			
Das et al., 2017	18:16	32.77	NMES on QC F: 30–75 Hz DC (on/off): 10s/50s PD: 20–1000 μs D: 30 min + exercises protocol	Exercises program: warm up exercises, flexibility exercises, strengthening exercises in WBP and non-WBP	Three sessions/week, four weeks	Pain	VAS10	One week	4	Significant differences favoring the SG were found regarding VAS, MVIC, VMO: VL ratio, and KPS 4th week post-intervention (P = 0.003, 0.045, 0.002, 0.001) respectively
						Function	KPS			
						Strength	HHD			
						Muscle Activity	VMO: VL ratio EMG			
Bily et al., 2008	19:19	25.35	EMS on QC F: 40 Hz DC (on/off): 5s/10s D: 20 min + exercises protocol	Exercises isometric, concentric, & excentric leg raises and pulls, stepping, squatting and balance exercises	two sessions/day, five days/week, 10 weeks	Pain	VAS10	Three months: one year	One at three months and five at one year	There is no statistically significant difference between the two groups in all outcome measures
						Function	KPS			
						Strength	Strain gauge			

**D**: Duration, **DC**: Duration Cycle, **EMG**: Electromyography, **EMS**: Electrical Muscle Stimulation, **F**: Frequency, **GMED**: Gluteus Medius, **HHD**: Hand-held Dynamometer, **HVPGS**: High Voltage Pulsed Galvanic Stimulation, **iEMG**: Integrated Electromyography, **KPS**: Kujala Patellofemoral Scale, **LESF**: Lower Extremity Functional Scale, **MVIC**: Maximum Voluntary Isometric Contraction, **NA**: Not Available, **NMES**: Neuromuscular Electrical Stimulation, **PD**: Pulse Duration, **PENS**: Patterned Electrical Neuromuscular Stimulation, **PPSC**: Patellofemoral Pain Severity Scale, **QC**: Quadriceps, **RMS**: Root Mean Square, **SG**: Study Group, **VAS**: Visual Analogue Scale, **VL**: Vastus Lateralis, **VMO**: Vastus Medialis Obliques, **WBP**: Weight-bearing Position

**Fig. 2:**
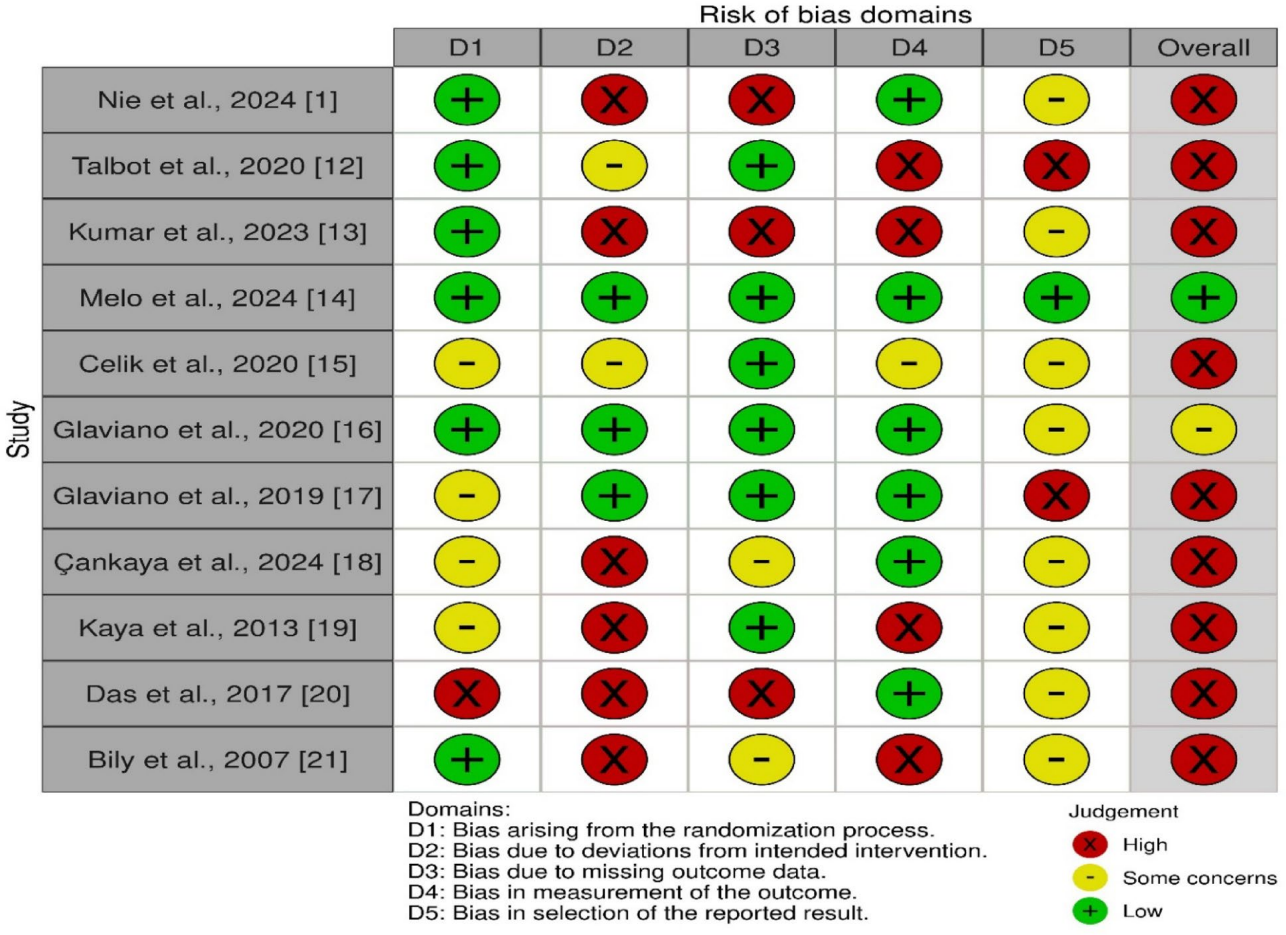
Risk of bias assessment score

According to ROB 2.0, Nine of the included studies were classified as having a high risk of bias [1, 19–21, 23–27], one study had some concerns [22] and one study was deemed to have a low risk of bias [20], The details of the risk of bias assessment are presented in Figure 2. (ROB). Also, the GRADE assessment of evidence certainty showed that all pooled outcomes were of very low certainty Table 2.

**Table 2:** Assessment of certainty of evidence

Certainty assessment							No of patients		Effect	Certainty	Importance
No of studies	Study design	Risk of bias	Inconsistency	Indirectness	Imprecision	Publication Bias	NMES	Control	Absolute (95% CI)		
**Pain (< 6w: assessed with VAS and PPSS)**											
3	RCT	Very serious^a^	Serious^b^	Not serious	Serious^c^	None	62	64	SMD **0.22 SD lower** (1.07 lower to 0.63 higher)	⨁◯◯◯ Very low	CRITICAL
**Pain (≥ 6w: assessed with VAS)**											
4	RCT	Very serious^a^	Not serious	Not serious	Serious^c^	None	94	94	MD **2.72 lower** (5.66 lower to 0.22 higher)	⨁◯◯◯ Very low	CRITICAL
**Function (< 6w: assessed with AKPS)**											
2	RCT	Very serious^a^	Serious^b^	Not serious	Serious^c^	None	29	30	MD **2.15 higher** (7.04 lower to 11.34 higher)	⨁◯◯◯ Very low	CRITICAL
**Function (≥ 6w: assessed with AKPS)**											
4	RCT	Very serious^a^	Not serious	Not serious	Serious^c^	None	75	73	MD **3.74 higher** (1.68 higher to 5.81 higher)	⨁◯◯◯ Very low	CRITICAL
**Quadriceps Strength (< 6w: assessed with handheld dynamometer)**											
2	RCT	Very serious^a^	Not serious	Not serious	Serious^c^	None	48	49	SMD **0.24 SD higher** (0.16 lower to 0.64 higher)	⨁◯◯◯ Very low	IMPORTANT
**Quadriceps Strength (≥ 6w: assessed with handheld dynamometer and Strain gauges)**											
3	RCT	Very serious^a^	Not serious	Not serious	Serious^c^	None	65	65	SMD **0.53 SD higher** (0.18 higher to 0.89 higher)	⨁◯◯◯ Very low	IMPORTANT

**CI**: confidence interval; **MD**: mean difference; **SMD**: standardized mean difference

^a^Crucial limitations sufficient to substantially lower confidence in the estimate of effect

^b^ Inconsistency: Serious, I2 > 50%

^c^sample size less than 800 x

## Primary outcomes

### Effect of NMES on pain

Ten included studies [1, 18–20, 22–27] examined the effect of NMES on pain using visual analogue scale (VAS) [1, 18, 19, 22, 23, 25–27], patellofemoral pain severity scale (PSS) [24] and numerical pain rating scale (NPS) [20].

**Fig. 3:**
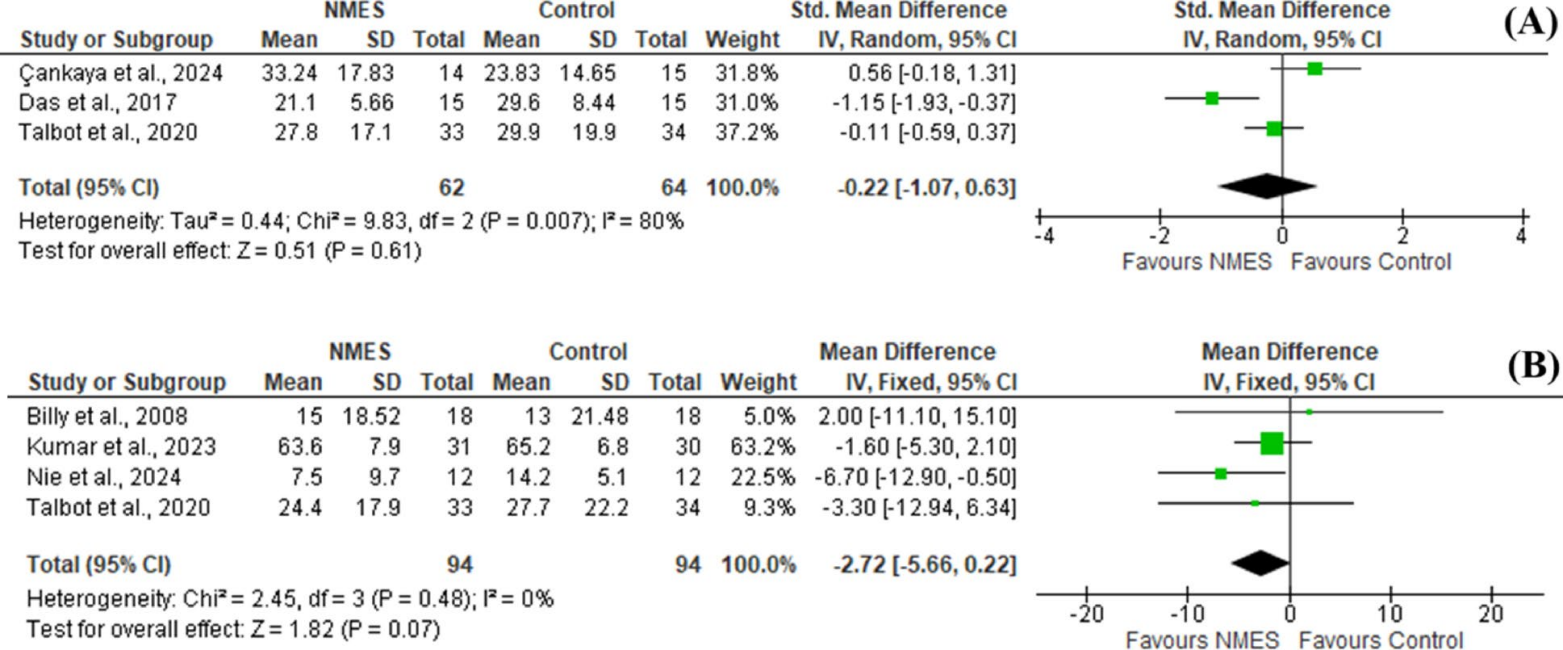
Forest plot of comparison: Comparison between NMES and control groups, outcome: (**A**) Pain in low and medium frequency NMES (< 6w) and (**B**) Pain in low frequency NMES (6 weeks or more)

Pooled statistical analysis of six studies [1, 18, 19, 24, 26, 27] revealed no significant difference between groups regarding pain intensity either in short (SMD = -0.22 [-1.07, 0.63], *P* = 0.61) or in the long courses of treatment (SMD =-2.72 [-5.75, 0.22], *P* = 0.48) (Fig. 3).

### Effect of NMES on muscle strength

**Fig. 4:**
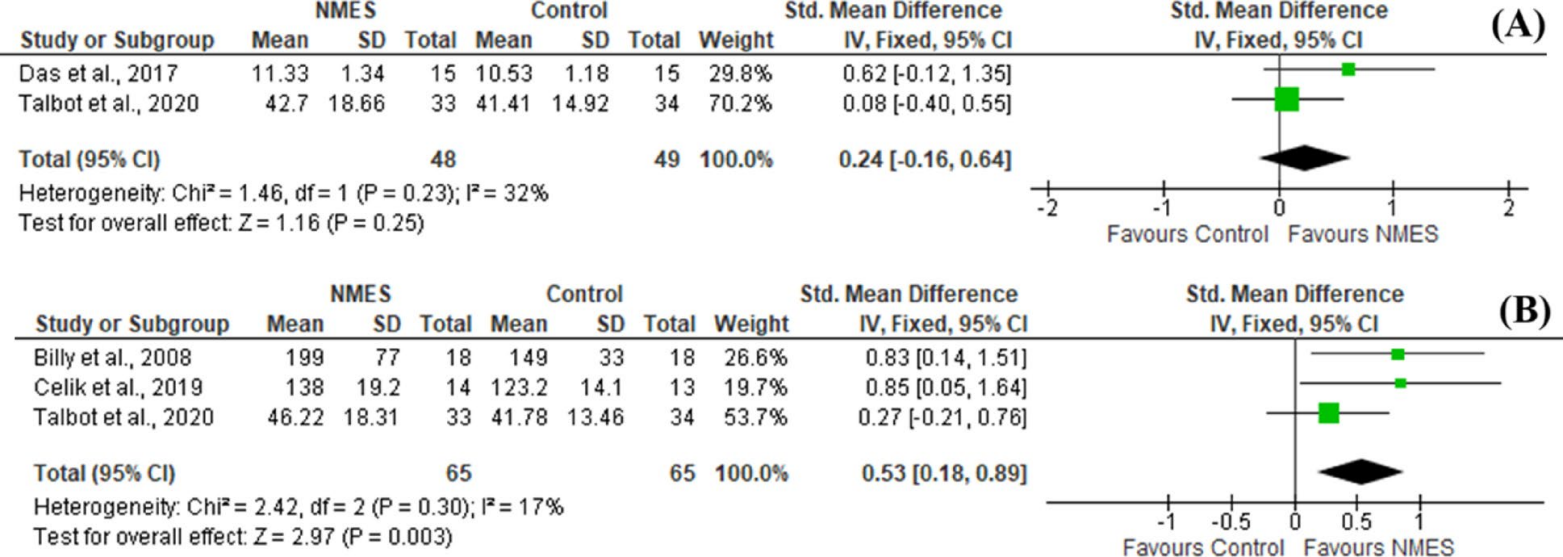
Forest plot of comparison: 1 Comparison between NMES and control groups, outcome: (**A**) Quadriceps strength in low frequency NMES (less than 6 weeks) (**B**) Quadriceps strength in low frequency NMES (6 weeks or more)

Muscle strength was assessed in six studies via isokinetic dynamometer [20, 21], handheld dynamometer [18, 23, 26] or strain gauge [27]. Pooled statistical data of two studies [18, 26] revealed no significant differences between groups in the short treatment course (SMD = 0.24 [-0.16, 0.64], *P* = 0.25), however, in the long-treatment course assessment, pooled statistical analysis of three studies [18, 21, 27] showed statistically significant difference in favor of NMES plus exercise compared to exercise alone (SMD = 0.53 [0.18, 0.89], *P* = 0.003) (Fig. 4).

### Effect of NMES on function

Knee function of individuals with PFP was assessed in eight studies using Kujala anterior knee pain scale (KPS) [1, 19–21, 23, 24, 26, 27]. Other measurement of knee function included the stair climbing test [24], lysholm scale [21] and the lower extremity function scale [23].

**Fig. 5:**
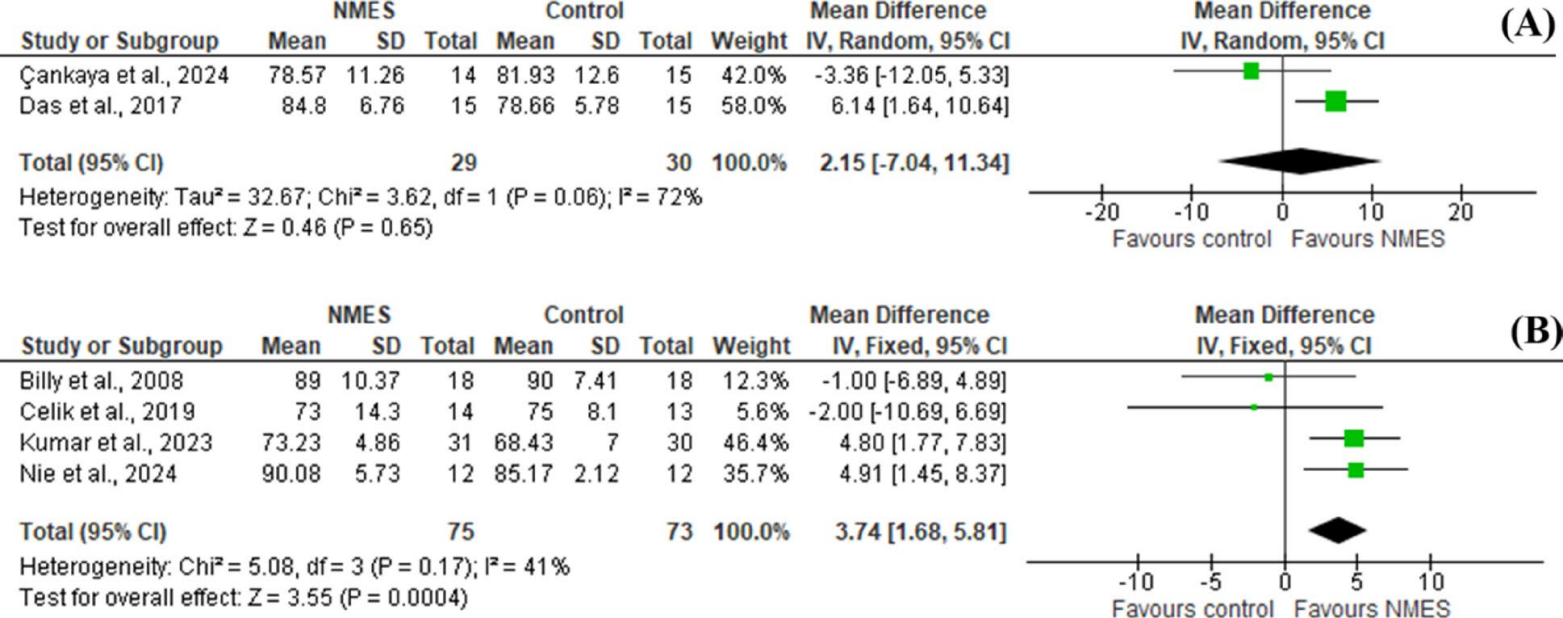
Forest plot of comparison: 1 Comparison between NMES and control, outcome: (**A**) Function via KPS in low and medium frequency NMES (< 6w) (**B**) Function via KPS in low frequency (6w or more)

Pooled statistical analysis of two studies [24, 26] showed no statistically significant difference between groups on the short treatment course (SMD = 2.15 [-7.04,11.34], *P* = 0.65) however, on long course of treatment, pooled data from four studies [1, 19, 21, 27] revealed significant difference in favor of NMES plus exercise compared to exercise alone (SMD = 3.74 [1.35, 5.81], *P* = 0.0004) (Fig. 5).

## Secondary outcomes

### Effect of NMES on muscle activity

Muscle activity was assessed via EMG in four studies [1, 20, 22, 26] reporting different EMG variables mostly vastus medialis obliques to vastus lateralis ration (VMO/VL ratio) and root mean square of knee muscles activity. Results were conflicting across studies with two studies [1, 26] reporting significant differences in favor of NMES while the other two showed no significant difference between groups [20, 22].

### Effect of NMES on activity and participation

Individuals with PFP participation and activity were evaluated in two studies [23, 24] through assessing their performance of activities of daily living via knee outcome survey-activities of daily living scale, activities of daily living scale, FitBit band activity tracker, fear-avoidance beliefs questionnaire. Both studies reported no significant differences between groups in all the measurement scales and tools.

**Fig. 6:**
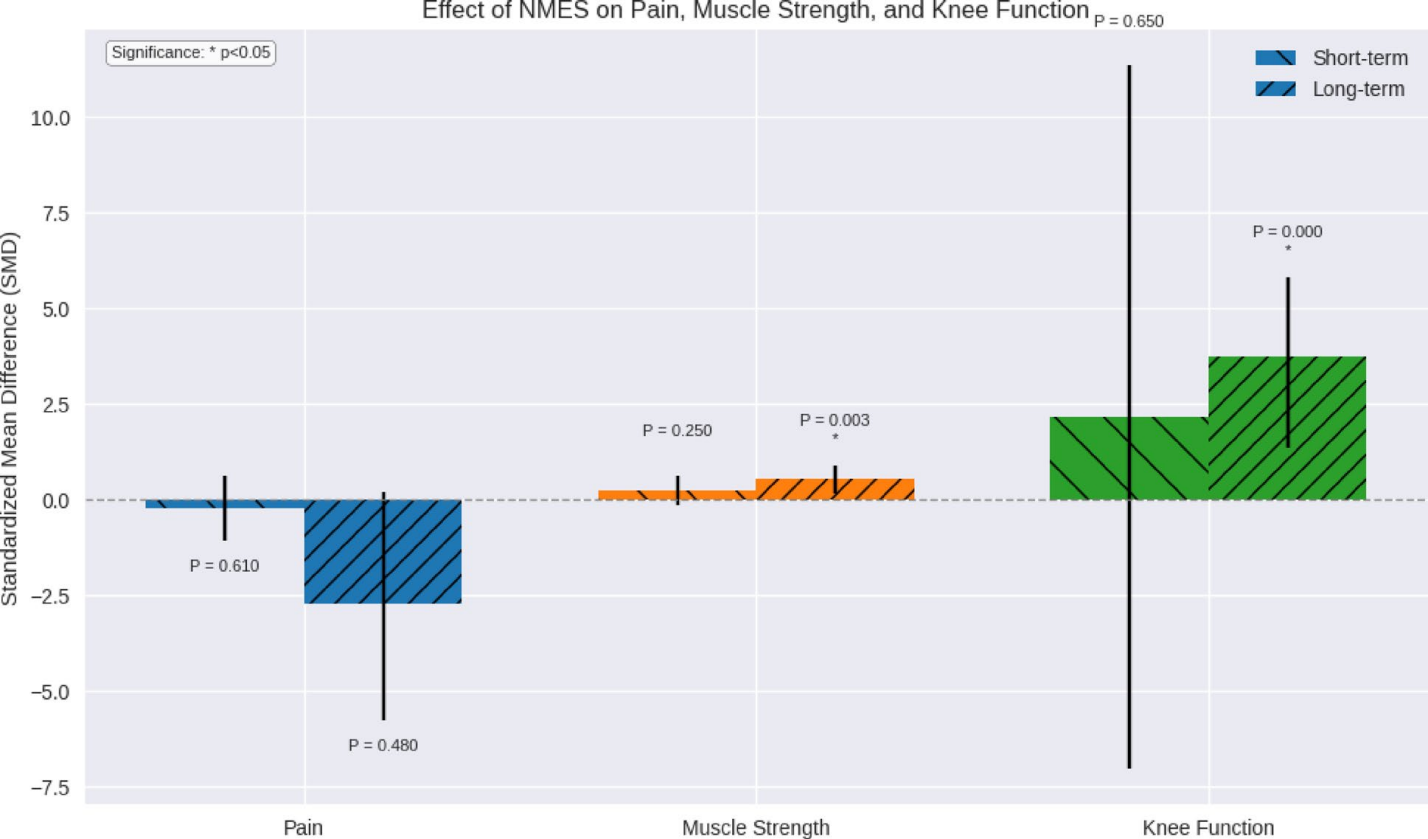
Summary of the Effectiveness of NMES on Pain, Muscle Strength, and Knee Function

The results are summarized in Fig. 6 showing the statistical significance of NMES efficacy in the three primary investigated outcomes.

## Discussion

This systematic review investigated the efficacy of NMES combined with exercise on pain, function, muscle strength, muscle activity and activity and participation in patients with PFP. Very low-quality evidence suggests that NMES combined with exercise may significantly improve function and quadriceps muscle strength at the long course of treatment compared to exercise alone; however, NMES combined with exercise do not differ from exercise alone in all other outcomes in both short and long course of treatment evaluation.

These results align with a systematic review conducted by Li and his colleagues [33], which found that long-term postoperative rehabilitation using neuromuscular electrical stimulation (NMES) after ACL surgery significantly, enhanced quadriceps muscle strength compared to standard rehabilitation alone. Additionally, a previous meta-analysis supported these findings [34]. However, some studies have raised questions about these results [35, 36].

These results suggest that the combined intervention effectively restored quadriceps activation and strength to levels similar to those of healthy adults. This improvement may be due to enhanced recruitment and firing of alpha motor neurons, which leads to greater voluntary activation [37].

Type II muscle fibers are believed to be selectively inhibited following a knee injury [38]. However, certain exercises can enhance quadriceps activation by promoting the selective recruitment of these Type II muscle fibers [39]. Moreover, neuromuscular electrical stimulation (NMES) tends to reverse the typical order of motor unit recruitment seen during voluntary contractions, giving preference to fast-twitch muscles with larger fiber areas. Therefore, it is reasonable to consider that training programs incorporating NMES may improve synchronization during muscle actions, particularly in fast-twitch fibers, ultimately leading to enhanced muscular power [40].

Regarding pain intensity, the results showed no statistically significant difference between groups, either for the intervention of less than six weeks or for the intervention of more than six weeks. These results can be explained by the fact that NMES forms had a greater impact on functional measures than on reported pain. This is supported by a comparative review conducted by Allen and others, who mentioned that some forms of ES addressing the recovery of muscle or nerve dysfunction do not simultaneously provide pain relief [41].

In line with other researchers, NMES has been found effective in rehabilitating spinal cord injury and immobilized/debilitated inpatients, as well as cerebral palsy patients. However, it does not appear to significantly reduce pain [3, 42]. Moreover, the effectiveness of NMES for pain relief is still uncertain. There have been some reports of reduced neck pain in patients with cervical spondylosis compared to sham treatment, however, most clinical studies are of low quality [3, 43].

Another possible explanation for the results obtained could be that none of the studies included in the analysis utilized neuromuscular electrical stimulation at either low frequency (less than 10 Hz) or higher frequency (100 Hz), as both of which could potentially induce an analgesic effect [44, 45].

The low-frequency TENS is applied with a frequency of less than 10 Hz and strong intensity, which elicits muscle contraction and depolarize Aδ and C fibers resulting in pain reduction through activating descending pain-modulating mechanisms originating from the brain stem On the other hand, 100-Hz elector-acupuncture triggers the release of serotonin through descending fibers which stimulates the release of spinal enkephalin resulting in inhibition of noxious inputs [44–47].

Concerning the impact of PFP on muscle strength and knee function, it was noted that quadriceps strength is a significant predictor of subjective function when assessed by the ADLS in patients with PFP. The study conducted by Glaviano & Saliba found a strong relationship between self-reported function and lower-extremity strength [48].

Furthermore, NMES can induce exercise-like molecular effects that potentially can lead to health and performance benefits in individuals who are unable to perform resistance exercise [49]. After an initial 6 weeks of training, increases in the cross-sectional area of the triceps brachii and pectoralis major muscles and maximum isometric voluntary contraction of the elbow extensors were reported [50]. Similarly, Munoz-Martinez and his colleagues reported that to achieve large-magnitude improvements in maximum oxygen uptake, 14–30 sessions for 6–12 weeks are recommended [51].

The results align with those of the current systematic review, showing no significant overall effect size of quadriceps strength in low-frequency NMES (less than 6 weeks). However, there was a significant overall effect on the size of quadriceps strength in low-frequency NMES (more than 6 weeks). Moreover, it appears that NMES can effectively increase muscle thickness, regardless of the frequency or the type of muscle being studied [52, 53].

When explaining increases in maximal strength, literature offers various explanatory approaches such as functional, morphological, and neuronal adaptations [54]. Goldspink and his colleague described the number of parallel sarcomeres (muscle cross-sectional area) to be a potential predictor for augmentation of muscle strength [55].

Considering that most of the included studies applied NMES to the quadriceps muscle, specifically the VMO [1, 18–21, 24, 25, 27], several potential factors could contribute to the increase in the thickness of the VMO muscle. The NMES alone appears to effectively contribute to the improvement of patients’ performance, potentially attributed to its ability to increase thigh cross sectional area, and range of motion [56, 57].

Finally, based on previous research, NMES leads to alterations in the motor recruitment process. This leads to increased activation of muscle fibers, especially type II muscles involved in forceful contractions, synchronization of motor unit activity, enhanced synaptic transmission, increased fiber excitability, and overall improvement in motor control [58–60].

## Limitations

Several limitations should be acknowledged when interpreting our findings. A key limitation is the small number of the included studies (*n* = 11) and the limited sample sizes (*n* = 571), which hinders the generalizability of our findings, in addition, the high risk of bias and heterogeneity in the intervention characteristics across most of the included studies were major concerns during conducting this review. Also, publication bias was not assessed in the present study due to the limited number of the included studies. Therefore, the results of this review should be approached with due caution.

## Future implications

The current systematic review revealed very low to low evidence supporting the use of NMES combined with exercise for improving knee function and quadriceps muscle strength with long course of treatment in patients with PFP. However, several aspects remain unexplored and require further clarification in future investigations. Due to the wide variety of parameters between the NMES trials and the short-term effects addressed in our results make it challenging to reach definitive conclusions. Additionally, the recommendations are based on very low- certainty evidence, Emphasizing the need for high-quality research, larger sample size, and consistent parameter between trials in the administrated intervention program to facilitate knowledge translation.

## Conclusion

Very low certainty of evidence suggests that adding NMES to an exercise program may possibly improve the knee joint function and quadriceps strength with treatment courses of six weeks or more in patients with PFP. However, it is not superior to traditional physical therapy programs in relieving pain, improving EMG activity, activity and participation. The current quality of evidence is limited. Thus, further high-quality RCTs with larger sample sizes and longer follow-up periods are needed for stronger evidence.

## Supplementary Information

Below is the link to the electronic supplementary material.

Supplementary Material 1

Supplementary Material 2

Supplementary Material 3

Supplementary Material 4

## Data Availability

No datasets were generated or analysed during the current study.
