# Supplementary material for: The effectiveness of neuromuscular electrical stimulation on pain, function, and quadriceps muscle strength in adults with patellofemoral pain: A systematic review and meta-analysis
Source: BMC Musculoskelet Disord. 2025 Aug 9;26:770. doi: 10.1186/s12891-025-09029-5 (PMC12335092; doi:10.1186/s12891-025-09029-5)
Supplement: Supplementary file 1 — Supplementary Material 1 [file 12891_2025_9029_MOESM1_ESM.docx]

**Title:** Effect of Neuromuscular Electrical Stimulation on Patients with Patellofemoral Pain Syndrome: A Systematic Review

**Search Date:** 23^rd^ July 2024

**Results:**

| Database | Search Term | N |
| --- | --- | --- |
| PubMed | (("patellofemoral pain"[Title/Abstract]) OR ("patello-femoral pain"[Title/Abstract])) OR ("anterior knee pain"[Title/Abstract]) AND (("electrical stimulation"[Title/Abstract]) OR ("electric neuromuscular stimulation"[Title/Abstract]) OR (Russian[Title/Abstract]) OR ("High Voltage Pulsed Galvanic stimulation"[Title/Abstract]) OR ("electrical nerve stimulation"[Title/Abstract])) | 43 |
| Scopus | ( TITLE-ABS-KEY ( "patellofemoral pain" ) OR TITLE-ABS-KEY ( "patello-femoral pain" ) OR TITLE-ABS-KEY ( "anterior knee pain" ) AND TITLE-ABS-KEY ( "Electrical stimulation" ) OR TITLE-ABS-KEY ( "Electrical neuromuscular stimulation" ) OR TITLE-ABS-KEY ( "electric muscle stimualtion" ) OR TITLE-ABS-KEY ( russian ) OR TITLE-ABS-KEY ( "High Voltage Pulsed Galvanic Stimulation" ) OR TITLE-ABS-KEY ( "Electrical nerve stimulation" ) ) | 89 |
| WOS | (TS=("patellofemoral pain") OR TS=("patello-femoral pain") OR TS=("anterior knee pain")) AND (TS=("electrical stimulation") OR TS=(“electrical neuromuscular stimulation”) OR TS=(“electric muscle stimulation”) OR TS=(Russian) OR TS=("High Voltage Pulsed Galvanic Stimulation") OR TS=("electrical nerve stimulation”)) | 85 |
| Cochrane | #1 "patellofemoral pain" #2 "patello-femoral pain"  #3 "anterior knee pain" #4 "electrical stimulation" #5 “electrical neuromuscular stimulation” #6 “electric muscle stimulation” #7 Russian #8 "High voltage pulsed galvanic stimulation" #9 "Electrical nerve stimulation" #10 ((#1 OR #2 OR #3) AND (#4 OR #5 OR #6 OR #7 or #8 or #9)) | 42 |
| PEDro | “Patellofemoral pain” stimulation | 21 |
| Google Scholar | allintitle: "patellofemoral pain" stimulation | 48 |
| ClinicalTrials.gov | Condition/Disease: Patellofemoral Pain Syndrome Intervention: Electrical Stimulation | 12 |
| ICTRP | Patellofemoral AND Stimulation | 22 |
