# Supplementary material for: The effectiveness of neuromuscular electrical stimulation on pain, function, and quadriceps muscle strength in adults with patellofemoral pain: A systematic review and meta-analysis
Source: BMC Musculoskelet Disord. 2025 Aug 9;26:770. doi: 10.1186/s12891-025-09029-5 (PMC12335092; doi:10.1186/s12891-025-09029-5)
Supplement: Supplementary file 2 — Supplementary Material 2 [file 12891_2025_9029_MOESM2_ESM.docx]

| Reason for exclusion | Author |
| --- | --- |
| Undergoing. | NCT03184545 [1] |
| Inconsistent data reporting. | Akarcali et al. 2002 [2] |
| Not RCT | Garcia et al. 2010 [3] |
| Combined effect of electrical stimulation & cryotherapy and exercises and control group take only exercises | Roh et al. 2021 [4] |
| Immediate effect (only one session) | Glaviano et al. 2016 [5] |
| Immediate effect (only one session) | Glaviano et al. 2016 [6] |
| All groups take electrical stimulation. (ineligible control) | Talbot et al. 2023 [7] |
| Thesis only | Sadeh et al. 2018 [8] |
| All groups take electrical stimulation. (ineligible control) | Callaghan et al. 2001 [9] |
| All groups take electrical stimulation. (ineligible control) | Callaghan et al. 2004 [10] |
| Not RCT | KURU et al. 2012 [11] |
| Written in Chinese | Jing et al. 2024 [12] |
| Different type of interventions | Samakosh et al. 2024 [13] |

Appendix (2) Sample of excluded articles with reasons for exclusion and reference

Excluded articles bibliography

[1] NCT03184545. Comparing the Outcome of Electrical Stimulation and Physical Therapy in Patellofemoral Pain Syndrome. Https://ClinicaltrialsGov/Show/NCT03184545 n.d. https://doi.org/10.1002/CENTRAL/CN-01494912.

[2] Akarcali I, Tugay N, Kaya D, Atay A, Doral MN. The role of high voltage electrical stimulation in the rehabilitation of patellofemoral pain. Pain Clin 2002;14:207–12. https://doi.org/10.1163/156856902320761397.

[3] Garcia FR, Azevedo FM, Alves N, Carvalho AC, Padovani CR, Negrão Filho RF. Effects of electrical stimulation of vastus medialis obliquus muscle in patients with patellofemoral pain syndrome: an electromyographic analysis. Rev Bras Fisioter 2010;14:477–82. https://doi.org/10.1590/S1413-35552010000600005.

[4] Roh Y, Park J. Training and Detraining Effects of a Rehabilitation Program with or without Electro-Cryotherapy in Patients with Anterior Knee Pain: A Randomized Trial. Appl Sci 2021;11:4812. https://doi.org/10.3390/app11114812.

[5] Glaviano NR, Saliba SA. Immediate Effect of Patterned Electrical Neuromuscular Stimulation on Pain and Muscle Activation in Individuals With Patellofemoral Pain. J Athl Train 2016;51:118–28. https://doi.org/10.4085/1062-6050-51.4.06.

[6] Glaviano NR, Huntsman S, Dembeck A, Hart JM, Saliba S. Improvements in kinematics, muscle activity and pain during functional tasks in females with patellofemoral pain following a single patterned electrical stimulation treatment. Clin Biomech 2016;32:20–7. https://doi.org/10.1016/j.clinbiomech.2015.12.007.

[7] Talbot LA, Webb L, Morrell C, Enochs K, Hillner J, Fagan M, et al. Electromyostimulation With Blood Flow Restriction for Patellofemoral Pain Syndrome in Active Duty Military Personnel: A Randomized Controlled Trial. Mil Med 2023;188:e1859--e1868. https://doi.org/10.1093/milmed/usad029.

[8] Sadeh S, Jensen JL, Kirn H. Dynamic Gait-Synchronous Neuromuscular Electrical Stimulation of Quadriceps in Patellofemoral Pain Patients. Arch Phys Med Rehabil 2018;99:e87--e88. https://doi.org/10.1016/j.apmr.2018.07.312.

[9] Callaghan MJ, Oldham JA, Winstanley J. A comparison of two types of electrical stimulation of the quadriceps in the treatment of patellofemoral pain syndrome. A pilot study. Clin Rehabil 2001;15:637–46. https://doi.org/10.1191/0269215501cr457oa.

[10] Callaghan MJ, Oldham JA. Electric muscle stimulation of the quadriceps in the treatment of patellofemoral pain11A commercial party with a direct financial interest in the results of the research supporting this article has conferred or will confer a financial benefit on the autho. Arch Phys Med Rehabil 2004;85:956–62. https://doi.org/10.1016/j.apmr.2003.07.021.

[11] Kuru T. Comparison of efficiency of Kinesio® taping and electrical stimulation in patients with patellofemoral pain syndrome. Acta Orthop Traumatol Turc 2012;46:385–92. https://doi.org/10.3944/AOTT.2012.2682.

[12] Jing W, Yingce Y, Xiaowei Y, Boshi X, Jianbin Z, Chen Y, et al. Intervention of muscle strength training combined with neuromuscular electrical stimulation on lower limb function and biomechanical changes in patients with patellofemoral pain. Chinese J Tissue Eng Res 2024;28. <https://doi.org/10.12307/2024.036>.

[13] Mohammadi Nia Samakosh H, Oliveira R, Shahabi S, Sarvarifar B, Moddares Gorji S, Amirkhanloo A, et al. Effects of High-intensity Training and Electrical Stimulation on Pain, Disability, Knee Kinematic and Performance in Patellofemoral Pain: A Randomized Controlled Trial. Retos 2024;55:978–91. https://doi.org/10.47197/retos.v55.105913.
